# Modifications to glucose-6-phosphate dehydrogenase for industrial applications: predictions and tests

**DOI:** 10.1186/1758-2946-5-S1-O19

**Published:** 2013-03-22

**Authors:** Martin Mosisch, Jan Simons, Lutz Hilterhaus, Andrew Torda

**Affiliations:** 1 Biomolecular Modelling, University Hamburg, Hamburg, Germany; 2 Institute of Technical Biocatalysis, Technical University Hamburg, Hamburg - Harburg, Germany

## 

We have been combining computational methods with experimental tests to gently modify glucose-6-phosphate dehydrogenase (G6PDH) from Leuconostoc mesenteroides for two specific goals. Firstly, it should be immobilized on an inorganic substrate as part of a longer-term project involving microreactor methods. Secondly, the natural enzyme is found as a homodimer, but we would like to have active monomers which would be better suited to the use of small pores in an aerogel based-system.

Our approach has involved consideration of available conservation information, structural properties and simple methods for estimating free energy changes. The approach relies completely on the crystal structure deposited in the protein data bank [1].

Covalent binding to a fixed surface involves introducing side-chains with appropriate reactive groups such as the thiols in cysteines, but there is some choice as to groups which can be incorporated into the inorganic surface. Here, the only considerations have been surface accessibility, distance from the active site and evolutionary conservation. One would rather substitute a naturally variable residue than a conserved site.

The second aim is more ambitious and speculative. The enzyme is naturally homo-dimeric, but a monomer might be easier to incorporate in the pores of the substrate. The approach here has been to identify sites at the monomer-monomer interface. From this small set of residues, a crude, but fast free energy estimator [2] was used to check substitutions which should destabilize the dimer while not destabilizing the monomer. First results from analytical chromatography and light scattering suggest a substantial monomer population with activity reduced by perhaps two orders of magnitude.

## References

[B1] Rowland P Basak AK Gover S Levy HR Adams MJ The three-dimensional structure of glucose 6-phosphate dehydrogenase from Leuconostoc mesenteroides refined at 2.0 A resolution Curr Biol 1994 2 1073 1087 10.1016/s0969-2126(94)00110-3 7881907

[B2] Schymkowitz J Borg J Francois S Nys R Rousseau F Serrano L The FoldX web server: an online force field Nucl Acids Res 2005 33 382 388 10.1093/nar/gki387 PMC1160148 15980494

